# The benefit of repetitive skills training and frequency of expert feedback in the early acquisition of procedural skills

**DOI:** 10.1186/s12909-015-0286-5

**Published:** 2015-02-19

**Authors:** Hans Martin Bosse, Jonathan Mohr, Beate Buss, Markus Krautter, Peter Weyrich, Wolfgang Herzog, Jana Jünger, Christoph Nikendei

**Affiliations:** 1 General Pediatrics, Neonatology and Pediatric Cardiology, University Children’s Hospital, Düsseldorf, Germany; 2 Department of General Internal and Psychosomatic Medicine, University of Heidelberg Medical Hospital, Im Neuenheimer Feld 410, 69120 Heidelberg, Germany; 3 Department of Nephrology, University of Heidelberg, Heidelberg, Germany; 4 University Department of Medicine, Department of Internal Medicine IV, Endocrinology and Diabetology, Angiology, Nephrology and Clinical Chemistry, University of Tübingen, Tübingen, Germany

**Keywords:** Cognitive workload, Procedural skills acquisition, Expert feedback, Learning curve, Nasogastric tube, Proficiency level, Skills training, Undergraduate medical education, Simulation

## Abstract

**Background:**

Redundant training and feedback are crucial for successful acquisition of skills in simulation trainings. It is still unclear how or how much feedback should best be delivered to maximize its effect, and how learners’ activity and feedback are optimally blended. To determine the influence of *high-* versus *low-frequency* expert feedback on the learning curve of students’ clinical procedural skill acquisition in a prospective randomized study.

**Methods:**

N = 47 medical students were trained to insert a nasogastric tube in a mannequin, including structured feedback in the initial instruction phase at the beginning of the training (T_1_), and either additional repetitive feedback after each of their five subsequent repetitions (high-frequency feedback group, HFF group; N = 23) or additional feedback on just one occasion, after the fifth repetition only (low-frequency feedback group, LFF group; N = 24). We assessed a) *task-specific clinical skill performance* and b) *global procedural performance* (five items of the *Integrated Procedural Performance Instrument* (IPPI); on the basis of expert-rated videotapes at the beginning of the training (T_1_) and during the final, sixth trial (T_2_).

**Results:**

The two study groups did not differ regarding their baseline data. The calculated ANOVA for *task-specific clinical skill performance* with the between-subject factor ‘Group’ (HFF vs. LFF) and within-subject factors ‘Time’ (T_1_ vs. T_2_) turned out not to be significant (p < .147). An exploratory post-hoc analyses revealed a trend towards a superior performance of HFF compared to LFF after the training (T_2_; p < .093), whereas both groups did not differ at the beginning (T_1_; p < .851). The smoothness of the procedure assessed as *global procedural performance*, was superior in HFF compared to LFF after the training (T_2_; p < .004), whereas groups did not differ at the beginning (T_1_; p < .941).

**Conclusion:**

Deliberate practice with both *high-* and *low-frequency* intermittent feedback results in a strong improvement of students’ early procedural skill acquisition. *High*-*frequency* intermittent feedback, however, results in even better and smoother performance. We discuss the potential role of the cognitive workload on the results. We advocate a thoughtful allocation of tutor resources to future skills training.

## Background

Skills lab facilities provide an effective and safe learning environment for undergraduate medical students to acquire clinical technical skills. Skills lab training leads to improved knowledge, skills, and behaviors when compared to standard clinical training or no training, with a moderate general effect for patient-related outcomes [1]. Skills lab training enables trainees to perform procedural skills faster, more accurately and more professionally on patients in terms of both technical and communicational aspects as compared to standard clinical training [2]. Furthermore, skills lab training leads to superior objective structured clinical examination (OSCE) results, both in longitudinal [3] and prospective controlled designs [4] even for long-time follow-up [5]. With regard to potential transfer, skills lab training sessions provide a better preparation for clinical clerkships [6] and result in a higher number of procedural skills being performed at bedside on wards [7]. A prerequisite for such transfer is to exercise great care in designing training models and scenarios in order to prove their validity with regard to the real clinical setting.

Four factors are described to enhance the learning of motor skills: observational practice, the learner’s focus of attention, feedback, and self-controlled practice [8]. Accordingly, in their Best Evidence Medical Education (BEME) guide, Issenberg et al. [9] identified similar factors leading to a maximum benefit of simulation-based medical education. The most relevant factors were a) *repetitive, active and standardized educational experiences*, to prevent trainees from being passive bystanders, b) *educational feedback*, and c) *embedding the training in the standard curriculum*. The majority of relevant articles found in their review agree on these three factors, and a plethora of data is available on the latter two. The fact that repetition and trainees’ activity per se are important factors to promote long-term retention is also unquestioned, both for low-complexity skills [10] and high-complexity skills [11].

Regarding learning curves in simulation-based education and motor-skills training, a dose-response relationship is assumed to exist, with a rising number of repetitions resulting in an increasingly superior performance until a performance plateau is reached [12,13]. So far, learning curves in skills lab training have been examined in different study populations for differing skills in various settings. For example, undergraduate students were shown to reach a plateau in endoscopic sinus surgery simulation or simulated peripheral venous cannulation after 5 to 10 trials [14,15], with fewer trials required as subjects progressed in their medical training. In a postgraduate clinical setting of anesthesia first-year residents, a rapid improvement of success for anesthesia procedures was observed during the first 20 attempts, leading to a success rate between 65 and 85% [12]. The model of Peyton seems to add an additional advantage in very early skill acquisition [5,16], serving as a springboard which enhances the benefit from subsequent repetitive practice. However, it remains unclear how subsequent practice should be optimally timed and designed.

Although most studies show feedback to be crucial for learning, many issues of how best to deliver feedback remain a matter of debate: *how much* feedback is required to attain a maximum benefit from repetitive skills training or to reach a proficiency level in the early acquisition of procedural skills; the ideal frequency, or mode of delivery of repetitive feedback; and how repetitions and feedback are optimally blended. Moreover, it is unclear whether repeating the feedback necessarily substantially improves performance at all [17-21].

Therefore, our randomized prospective study was designed to determine the influence of *high-* versus *low-frequency* expert feedback on the learning curve of students’ clinical procedural skill acquisition. Our hypothesis was that repetitive practice is beneficial for reaching a proficiency level, with an additional benefit when intermittent feedback is given at a higher frequency as opposed to intermittent feedback at a lower frequency.

## Methods

### Study design

The presented randomized prospective study investigated the influence of repetitive expert feedback in skills training on the learning curve of students in the early acquisition of procedural skills. Nasogastric tube placement was selected as the clinical task as this skill represents a pivotal routine procedure in internal medicine. If it is not performed accurately, severe complications may occur, resulting in considerable costs [22,23], and it is therefore an integral part of undergraduate skills training curricula [24,25]. The study was conducted over a period of two and a half weeks alongside the regular curriculum at our faculty.

### Participants

Trainees were recruited via advertisements among medical students in their first or second year of medical training. A total of N = 50 participants volunteered to participate. Only right-handed individuals were eligible for inclusion in order to standardize the camera position and facilitate raters’ assessment by not needing to change perspectives. Written consent was provided by all participants and data from all participants were treated anonymously. The trainees were informed that the purpose of the study was to assess their skill performance, but no further details were provided. All participants received a minor financial compensation for their study participation. Ethical principles according to the World Medical Association Declaration of Helsinki *Ethical Principles for Medical Research Involving Human Subjects* of 2008 were adhered to. Ethics approval was granted by the ethic committee of the University of Heidelberg (Nr. S-211/2009). Students with previous experience in inserting nasogastric tubes were excluded from the study. Refusal to participate had no impact on the subsequent evaluations or other assessments in the curriculum.

### Assessment prior to the training (T_0_)

To control for potential confounding variables, each study participant provided data on their age, sex, handedness, previous clinical experience regarding clerkships, qualification as a paramedic or nurse, as well as a general self-efficacy rating using the *General Self-Efficacy Scale* and individual learning styles using the *Kolb Learning Style Inventory (KLSI)* in order to prove that conditions in the two groups were equal.

#### General self-efficacy scale

This questionnaire consists of ten positively worded items rated on a four-point Likert scale (4 = “I agree” to 1 = “I disagree”). It assesses perceived self-efficacy in the event of adversity and stressful life events [26].

### Kolb Learning Style Inventory (KLSI)

The *Kolb Learning Style Inventory (KLSI)* of 2005 [27] showed an even distribution of learning styles as a potential confounder in skill acquisition although it’s immediate effect is unclear [28]. On the other hand, well-organized and strategic learning styles assessed with other inventories (which may be compared to the steps *reflective observation* and *abstract conceptualization* of the KLSI) have been shown to provide a benefit for students' later performance [29,30]. Additionally, learning settings should accommodate individual learning styles to maximize individual learning achievement [31] as learning styles not only differ but may shift across cognitive and motor settings [32].

### Assignment to study groups

**Figure 1:**
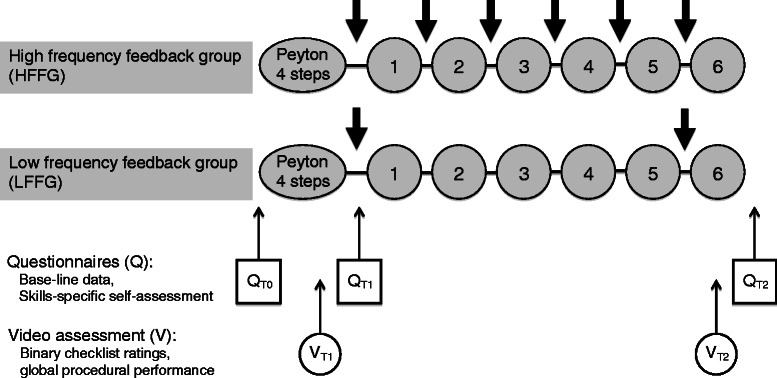
**Study design.** The study employed a randomized controlled design: high-frequency feedback group (HFF group, N = 23) and low-frequency feedback group (LFF group, N = 24). T_0_ assessment before training, T_1_ assessment after step 4 of Peyton and T_2_ assessment after the final repetition. Q assessment via questionnaire, V video assessment. The numbers indicate the six successive repetitions of inserting a nasogastric tube; the thick arrows indicate feedback given by the tutor. The assessment at T_0_ included questionnaires assessing a) *general self-efficacy rating*, b) the *Kolb Learning Style Inventory (KLSI)*, and c) *skill-specific self-efficacy ratings* (Q_T0_). The assessments at T_1_ and T_2_ included questionnaires assessing c) *skill-specific self-efficacy ratings* (Q_T1_ and Q_T2_), and d) objective video ratings of participants’ performances regarding *clinical performance* (binary checklists) and *overall procedural performance* (global rating; V_T1_ and V_T2_). In addition, the assessment at T_2_ included questionnaires assessing e) *acceptance*, f) *subjective skill-related demands* during skill performance, g) *value of feedback* (Q_T2_).

N = 50 participants were randomly assigned to one of the two study groups, one receiving high-frequency feedback (high-frequency feedback group, HFF group; N = 25) after each of the five repetitive practice trials before the final, sixth repetition, and one receiving low-frequency feedback only twice, i.e. after the first independent skill performance and just before the final, sixth repetition (low-frequency feedback group, LFF group; N = 25; for details see “skill training session” below; see Figure 1). After participants had been included in the study, three students opted not to participate without stating reasons or due to illness, resulting in a dropout of N = 2 in the HFF group and N = 1 in the LFF group. Thus, the final sample consisted of N = 23 in the HFF group and N = 24 in the LFF group.

### Skill training session

The skill training was conducted with a student-teacher ratio of 1:1 in analogy to previous studies [24,33] and under consideration of previously published checklists for nasogastric tube placement [24,34]. Training sessions were structured as follows: Both groups received a short case history and role-play directives (i.e. including talking to the mannequin as if it were a real patient), both of which have been previously shown to enhance perceived realism in the training as well as the patient-physician communication [24,33]. Both groups were then instructed to insert a nasogastric tube in a mannequin using the four steps of *Peyton’s Four-Step Approach* [16,35] and subsequently performed six further repetitions of inserting a nasogastric tube. Both groups received structured feedback from the skills lab trainers [9,36] after the first independent performance of nasogastric tube placement (step 4 of Peyton’s four-step approach; T_1_). However, the HFF group received performance-related feedback after each of their following five repetitions, whereas the LFF group received further feedback once, after the fifth repetition only. Finally, both groups performed a final, sixth repetition of nasogastric tube placement (T_2_; see Figure 1). The feedback was always given immediately after the respective repetition of the skill, as proximate feedback enhances its effectiveness [37]. Feedback was positively worded and aimed at inducing an external focus, i.e. aiming at the movement effect to facilitate automaticity in motor control and promote movement efficiency (for detailed reviews, see [8,38]).

### Skills lab teachers

Both the HFF and LFF group were both trained by four certified skills lab peer teachers, all of whom had at least one year of experience as skills lab trainers. Two tutors were male (both aged 22 years), and two were female (aged 21 and 22 years). The four tutors were randomly assigned to the students in the study groups. As previously shown, trained medical students as peer teachers deliver training and feedback on a par with that of faculty staff in skills training [39-41]. All trainers were blinded to the study design and received an introductory course including training in nasogastric tube placement and delivering feedback prior to the study.

### Assessment of training

Assessment of the skills lab training encompassed a) acceptance ratings including value of tutor’s feedback, b) subjective skill-related demands during skill performance (cognitive workload), c) skill-specific self-efficacy ratings related to nasogastric tube placement competencies, and d) objective video expert ratings of participants’ performances by blinded independent assessors (N = 2).

#### Acceptance ratings and value of trainer’s feedback

For the evaluation of acceptance of the training session and the tutor’s feedback, participants completed a questionnaire with five positively worded items rated on a six-point Likert scale (6 = fully agree; 1 = completely disagree) after completing the training (after T_2_, see Figure 1). For the specific pre- and post-evaluation of the value of the trainer’s feedback, the participants completed an additional questionnaire with ratings on a six-point Likert scale (6 = fully agree; 1 = completely disagree) after step 4 of Peyton’s four-step approach but before beginning the repetitions (T_1_, see Figure 1; 5 items) and after the final, sixth repetition (T_2_; 12 items).

#### Cognitive workload/skill-related demands

We assessed the perceived overall cognitive workload using the *National Aeronautics and Space Administration Task Load Index* (NASA-TLX) [42] as a score of six subscales: *mental*, *physical*, and *temporal demands*, as well as *own performance*, *effort* and *frustration*. Assessment took place after step 4 of Peyton’s four-step approach (T_1_) and after the final, sixth repetition (T_2_), with ratings on Likert scales from 5 (very low demands) to 100 (very high demands), resulting in a sum score between 0 (very low demands) and 100 (very high demands).

### Skill-specific self-efficacy ratings

*Skill-specific self-efficacy ratings related to nasogastric tube placement competencies* were assessed as in previous studies [33], with five items referring to a) knowledge of the anatomical structures required to insert a nasogastric tube, b) knowledge of the materials required to insert a nasogastric tube, c) knowledge of the steps involved in inserting a nasogastric tube, d) competence in inserting a nasogastric tube in a mannequin, and e) competence in inserting a nasogastric tube in a patient (6 = fully agree; 1 = completely disagree). Skill-specific self-efficacy ratings were assessed prior to the training (T_0_) as well as after step 4 of Peyton’s four-step approach (T_1_) and after the final, sixth repetition (T_2_).

#### Independent video assessment of performance

Participants’ performance in step 4 of Peyton’s four-step approach (T_1_) and in the final, sixth repetition (T_2_) were videotaped in both the HFF group and the LFF group using high-resolution digital cameras with optical zoom to capture all of the details required for a precise evaluation. The videos were digitally processed and were independently rated by two clinically experienced and trained video assessors who were blinded to both the aim and design of the study as well as the assignment of participants to the study groups. Raters were provided with binary checklists and global rating forms. The binary checklist consisted of 16 items reflecting the procedural steps of inserting a nasogastric tube [24,34,43]. Regarding binary checklists, video raters were asked to indicate whether single procedural steps were performed correctly or incorrectly. A global rating form, which was based on the *Integrated Procedural Performance Instrument* (IPPI) proposed by Kneebone et al. for the assessment of procedural skills in a clinical context, was also used [44]. The IPPI was designed to evaluate global professional and confident performance of clinical technical skills. Items of the IPPI considered relevant for the procedure were selected (items 4, 5, 9, 10, 11; six-point Likert scale; 6 = very good to 1 = unsatisfactory).

### Statistical analysis

Primary endpoint was the global procedural performance. Secondary endpoints were task-specific clinical skill performance, skill-specific self-efficacy ratings, and pre- and post-assessment of trainer’s feedback (T_1_ and T_2_). Data are presented as means and standard deviation. Continuous data serving sample description were compared using a Student’s t-test (assuming equal variances), whereas ordinal data were assessed using Mann-Whitney *U* test (M-W-U-Test)). Differences in group characteristics pertaining to sex, previous education in a medical profession, and completed medical electives were compared using chi-square tests. For repeated measures, ordinal data (acceptance ratings, cognitive workload assessed with NASA-LTX, skills-specific self-efficacy ratings, and global skills performance assessed with the IPPI) were calculated using Wilcoxon signed-rank test or Friedman test where appropriate. Group comparisons at T_1_ and at T_2_ were calculated using M-W-U-Tests. For interval data (task-specific clinical skills performance reflected in binary checklist ratings), an ANOVA with the between-subject factor ‘Group’ (HFF vs. LFF) and the within-subject factor ‘Time’ (T_1_ vs. T_2_) was conducted. LSD-post-hoc tests were used where appropriate. A p-value < .05 was considered statistically significant. Inter-rater reliability for the two video assessors was calculated using a Pearson’s-Correlation. The software package STATISTICA 8, 2007 (Statsoft, Inc., Tulsa, OK) was used for statistical analysis.

## Results

### Participants

There were no statistically significant differences between the two groups with regard to age, sex, completed education in a medical profession, or completed medical electives and general self-efficacy rating prior to skills training, as well as *learning styles* (see Table 1), with a distribution of subscales of learning styles as described previously [28].

**Table 1:** **Group characteristics of the study groups**

Group characteristics	High-frequency feedback group (HFF group) N = 23	Low-frequency feedback group (LFF group) N = 24	p-value
Age (years)	21.00 ± 2.94	20.62 ± 1.74	.596^1^
Sex (m/f)	12 (52.17%)/11 (47.82%)	12 (50.00%)/12 (50.00%)	.882^2^
General self-efficacy rating	30.83 ± 3.42	30.42 ± 4.15	.842^1^
Education in a medical profession	3 (13.04%)	1 (4.16%)	.276^2^
Medical electives	20 (86.95%)	21 (87.50%)	.955^2^
**Kolb learning style inventory**			
Concrete experience (*feeling*)	24.61 ± 7.65	24.79 ± 5.85	.927^1^
Reflective observation (*watching*)	30.30 ± 6.88	29.88 ± 5.54	.815^1^
Abstract conceptualization (*thinking*)	31.34 ± 8.61	32.37 ± 7.43	.663^1^
Active experimentation (*doing*)	33.30 ± 7.55	32.95 ± 5.28	.856^1^

^1^t-test.

^2^χ2 test.

Group characteristics of the high-frequency feedback group (HFF group, N = 23) and the low-frequency feedback group (LFF group, N = 24) are depicted regarding:

• age (age; mean ± standard deviation; Student’s t-test, p-values).

• sex (male/female; N and %, chi-square test, p-values).

• general self-efficacy rating prior to skills training (score of 10 items using Likert-scale ratings; 4 = I fully agree; 1 = I completely disagree; mean ± standard deviation; M-W-U-Test, p-values).

• completed education as paramedic, medical secretary, nurse, or occupational therapist.

• (N, % and chi-square test p-values).

• completed electives in surgery, internal medicine, pediatrics, or psychiatry (N, % and chi-square test, p-values).

### Acceptance of training (rated at T_2_) and assessment of trainer’s feedback (T_1_ and T_2_)

Participants of both study groups confirmed a high training acceptance after the skills training session. Participants rated the training session as realistic (HFF group 4.66 ± .98, LFF group 4.34 ± 1.13, p = .285) and the tutor’s feedback as objective (HFF group 5.96 ± .21, LFF group 5.96 ± .20, p = .992), motivating (HFF group 5.74 ± .45, LFF group 5.67 ± .76, p = .858), supportive (HFF group 5.96 ± .21, LFF group 5.92 ± .28, p = .825), and courteous (HFF group 6.0 ± .0, LFF group 6.0 ± .0, p = 0.992), with a positive effect on learning success (HFF group 6.0 ± .0, LFF group 5.92 ± .28, p = 0.635) all ratings are given as mean of Likert scale ratings from 6 = fully agree; 1 = completely disagree).

In the pre- and post-assessment, both groups assessed trainers’ feedback as very valuable after step 4 of Peyton’s four-step approach (T_1_) as well as after the final, sixth repetition (T_2_), with no significant difference between the study groups (see Table 2). Both groups assessed trainers’ feedback as more valuable after the final, sixth repetition (T_2_) compared to after step 4 of Peyton’s four-step approach (T_1_) (HFF p = .004; LFF p = .018).

**Table 2:** **Pre- and post-assessment of trainer’s feedback (T1 and T2) High-frequency feedback group**

	High-frequency feedback group (HFF group) N = 23	Low-frequency feedback group (LFF group) N = 24	p-value ^ 1 ^
*After Peyton’s step 4 (T1)*			
Item 1 *The tutor’s feedback was comprehensible*.	5.83 ± .38	5.84 ± .38	.931
Item 2 *I could easily follow the tutor’s instructions*.	5.39 ± .66	5.17 ± .96	.077
Item 3 *The feedback was helpful for improving skills*.	5.78 ± .42	5.79 ± .51	.382
Item 4 *The tutor was attentive and concentrated*.	5.83 ± .39	5.83 ± .38	.931
Item 5 *The tutor seemed competent during feedback*.	5.83 ± .39	5.87 ± .34	.518
Mean Items 1-5	5.73 ± .31	5.70 ± .46	.876
*After the final, sixth repetition (T2)*			
Item 1 *The tutor’s feedback was comprehensible*.	5.96 ± .21	6.00 ± .00	1.000
Item 2 *I could easily follow the tutor’s instructions*.	5.83 ± .39	5.58 ± .50	.224
Item 3 *The feedback was helpful for improving skills*.	5.91 ± .29	5.96 ± .20	.108
Item 4 *The tutor was attentive and concentrated*.	5.87 ± .34	5.92 ± .28	.351
Item 5 *The tutor seemed competent during feedback*.	6.00 ± .00	5.92 ± .28	1.000
Mean Items 1-5	5.91 ± .17	5.88 ± .19	.341
**p-value** ^**2**^	.004	.018	

^1^Mann Whitney U test.

^2^Wilcoxon signed rank test.

Perceived value of the feedback between the two groups after Peyton’s step 4 (T_1_), and after the final, 6th repetition (T_2_; mean and standard deviation of six-point Likert scales from 6 = fully agree to 1 = completely disagree; M-W-U-Test, p-values).

### Workload/skill-related demands perceived at T_1_ and T_2_

Both groups rated the overall skill-related demands as high, with no differences between the HFF and LFF group after step 4 of Peyton’s four-step approach (T_1_; HFF group 50.78 ± 12.0%; LFF group 47.15 ± 17.3%; p = .395; mean of scores from 0 = very low to 100 = very high using the *National Aeronautics and Space Administration Task Load Index*, NASA-TLX). After the final, sixth repetition (T_2_), both groups rated the task as less demanding compared to T_1_ (HFF group T_1_ 50.78 ± 12.02, T_2_ 40.51 ± 18.74, p.003; LFF group T_1_ 47.15 ± 17.35, T_2_ 35.97 ± 16.58, p = <.001), with no differences emerging between the HFF group and the LFF group at T_2_ (HFF 40.51 ± 18.74; LFF 35.97 ± 16.58; p = .407).

### Skill-specific self-efficacy ratings (T_0_, T_1_ and T_2_)

Self-efficacy ratings related to competencies regarding nasogastric tube placement improved substantially over the course of the training (HFF p < .001; LFF p < .001), but there was no significant difference between the study groups prior to the training (T_0_), after step 4 of Peyton’s four-step approach (T_1_) or after the final, sixth repetition (T_2_; see Table 3).

**Table 3:** **Skill-specific self-efficacy ratings**

	High-frequency feedback group (HFF group) N = 23	Low-frequency feedback group (LFF group) N = 24	MWU p-value ^ 1 ^
Prior to the training (T0)	2.35 ± .71	2.33 ± .64	.640
After Peyton’s step 4 (T1)	4.70 ± .59	4.86 ± .74	.296
After the final, sixth repetition (T2)	5.27 ± .39	5.37 ± .40	.872
**p-value** ^**2**^	<.001	<.001	

^1^Mann Whitney U test.

^2^Friedman test.

Self-efficacy ratings relating to five competencies in inserting a nasogastric tube between the two groups before the training (T_0_), after Peyton’s step 4 (T_1_), and after the final, 6th repetition (T_2_; mean and standard deviation of six-point Likert scales from 6 = fully agree to 1 = completely disagree; M-W-U-Test, p-values).

### Independent video assessment: task-specific clinical skill performance by expert binary checklist rating (T_1_, T_2_)

For expert binary checklist ratings, an ANOVA with the between-subject factor ‘Group’ (HFF vs. LFF) and within-subject factors ‘Time’ (T_1_ vs. T_2_) was conducted. The calculated ANOVA was not statistically significant for all effects (main effects and interaction; p = .147). Nevertheless, an exploratory post-hoc analysis was performed according to our predefined hypothesizes. As expected, during step 4 of Peyton’s four-step approach (T_1_), no difference was found between the HFF group and the LFF group in the total score for specific clinical skill performance (p = .851; see Table 4). At T_2_, both groups scored higher on *binary checklist ratings* compared to T_1_ (p < .001) with the HFF group showing higher scores at T_2_, although not significantly (p < .093; see Table 4).

**Table 4:** **Task-specific clinical skill performance and global procedural performance**

Task-specific clinical skill performance (binary checklists)			
	High-frequency feedback group (HFF group) N = 23	Low-frequency feedback group (LFF group) N = 24	p-value ^ 1 ^
Peyton’s step 4 (T1)	91.06 ± 7.48	91.42 ± 9.14	.851
Final, 6th repetition (T2)	99.22 ± 2.25	96.04 ± 4.96	.093
p value^1^	<.001	<.001	
**Global procedural performance (global rating)**			
	**High-frequency feedback group (HFF group) N = 23**	**Low-frequency feedback group (LFF group) N = 24**	**p-value** ^**2**^
Peyton’s step 4 (T1)	5.31 ± 0.50	5.30 ± .64	.941
Final, 6th repetition (T2)	5.95 ± 0.07	5.65 ± .48	<.004
p value^3^	<.001	.002	

^1^LSD-post-hoc Tests.

^2^Mann-Whitney-U Test.

^3^Wilcoxon signed-rank test (T1 vs. T2).

Performance ratings of the two groups in Peyton’s step 4 (T_1_) and in the final, 6th repetition (T_2_) in *task-specific clinical skill performance* (binary checklist rating as mean score in percent of maximum achievable points and standard deviation; checklist of 16 items with a minimum of 0 and a maximum of 16 points; ANOVA, p-values) and *global procedural performance* (global performance rating as mean score of global rating scales ± standard deviation; six-point Likert scale from 6 = very good to 1 = unsatisfactory; M-W-U-Test, p-values).

### Independent video assessment: global procedural performance rating (T_1_, T_2_)

Global ratings of procedural performance assessed with the *Integrated Procedural Performance Instrument* (IPPI) also revealed no difference at the beginning of the training during step 4 of Peyton’s four-step approach (T_1_; p = .941). At T_2_, both groups scored higher in their *global procedural performance compared to T1* (HFF group p < .001; LFF group p = .002). In addition, after the final, sixth repetition (T_2_), the HFF group achieving better scores than the LFF group (p = .004; see Table 4).

### Inter-rater reliability for independent video raters

Standardized inter-rater reliability for independent video raters was .79 for the assessment of step 4 of Peyton’s four-step approach, and .75 for the evaluation of the final, sixth repetition when using binary checklist ratings, and .76 for the assessment of step 4 of Peyton’s four-step approach, and .81 for the evaluation of the final, sixth repetition when using global performance ratings.

## Discussion

In this randomized prospective study, we assessed the influence of frequency of expert feedback during redundant practice in the early acquisition of students’ clinical procedural skills. Both *high-* and *low-frequency* intermittent feedback resulted in a significant improvement of students’ clinical procedural skill performance as primary endpoint, in line with earlier findings [1,2,4,45]. *High*-*frequency* intermittent feedback, however, proved to result in an even better procedural performance compared to *low-frequency* intermittent feedback. Regarding the exploratory analyses of task specific performance (as reflected by our binary checklist ratings), both groups benefited from the training but with no significant difference between the two groups. A limitation of our results is that high scores in skill performance were already found during the very first independent performance, which we attribute to the success of Peyton’s four-step framework of deconstruction and learners’ comprehension before actually performing the skill.

Regarding the investigated cohort, there was no difference between the two study groups prior to the experiment with respect to age, sex, prior medical education, number and field of chosen electives, general and skill-specific self-efficacy ratings relating to nasogastric tube placement, or individual learning styles. Pre- and post-skill-specific self-efficacy ratings were higher in both groups, with no significant difference. Although the correlation between self-efficacy – reflecting a modification of physicians’ perception, motivation, and activity [46] – and superior objective performance measures is called into doubt in the literature [47], previously published research implies that higher self-efficacy in skills training results in more rigorous demands by the students for supervision during the performance of clinical skills at bedside on patient wards [46-48]. Our results strengthen the findings that – irrespective of the frequency of intermittent feedback – repetitive deliberate practice represents one of the most important factors to enhance self-efficacy.

Taking a closer look at the learning curve (dependent on the complexity of the motor skill), both groups’ performance started from the same level at beginning of the training with relatively high scores after the first 4-step Peyton training, even before the first feedback (90%, see Table 4) and reaching a proficiency level of 95% after five consecutive repetitions. Thus we assume a medium degree of complexity compared to other settings [49-52].

The feedback of trainers aimed at aspects of trainees’ performance with better than average performance and the trainees’ very high rating of this feedback associated to their improved performance is in line with previous findings suggesting both informational and motivational influences of such feedback (for a comprehensive review, see [8]). As the two groups rated the value of trainers’ feedback equally high, we attribute effects of the study to the *variance in frequency* of feedback. Notably, more feedback, more frequent feedback, or even concurrent feedback, may not always necessarily have a positive impact [19] or even degrade learning [53]. In our study, we were able to show that *intermittent* feedback – at both low and high frequency – supports trainees’ performance in a complex motor skill.

This increase in performance among both study groups in terms of smoothness of the procedure (as reflected by our global rating scores), was more pronounced after a higher frequency of intermittent feedback. This is in contrast to other findings: Receiving no feedback in a surgical simulator setting led to more instrument smoothness than with receiving feedback – even though, as expected, more mistakes occurred [21]. Too intense feedback during the early stages of skill acquisition may even hinder learning [20]. This fact that reducing the frequency of feedback related to performance enhances motor skills learning has been described previously [54] and has not yet been satisfactorily explained. In our study high frequency intermittent feedback more affects the secureness and smoothness of the procedure to be performed, than the accurate performance of the single sub-steps (task-specific clinical skill performance).

### Limitations

Both groups already achieved high scores in their nasogastric skill performance during the very first independent performance, which could be attributed to the success of Peyton’s four-step framework of deconstruction and the learners’ comprehension before actually performing the skill. This only leaves small scope for improvement – but of note, there is still a significant effect for both groups. Notably, within Peyton’s four-step framework, there is no scheduled specific feedback. In line with previous findings, our undergraduates reached mastery after their sixth repetition. For more experienced members of the medical community, i.e. graduates, reduced or delayed feedback as well as a more complex instruction might be more successful [19].

We measure training performance during practice and during early stages of skills learning; currently, a final conclusion for retention and transfer of this performance to future practice is hard to draw. Our work group has addressed this apparent gap in literature previously; our setting seems to provide more potential for retention than the traditional bedside teaching [2,5].

## Conclusion

As a conclusion, the optimal benefit from feedback seems to be a question of timing and dosing. Regarding the pros and cons of frequency or timing of feedback, we were able to show that both *high-* and *low-frequency* intermittent positive feedback leave sufficient time for the learner for self-controlled practice as compared to continuous concurrent (= permanent) feedback [8]. Our intermittent feedback results in a positive impact on the on skill-specific self-efficacy and the learning curve of students’ clinical procedural skill acquisition, with a moderate reduction of cognitive workload over the training. In contrast, continuous concurrent (= permanent) feedback during skill acquisition may degrade the learning of skills: “It may be better to wait” [55] for a trainee to finish performing a defined sequence. In this sense, we conclude that both *low-frequency* and *high-frequency* positive feedback leave sufficient resources for the learner’s cognitive processing, with *high-frequency* positive feedback potentially being more demanding in this sense. However, apparently, this still leaves sufficient resources for this group to take advantage of their additional intermittent feedback in order to achieve mastery.
